# TIP-1 Translocation onto the Cell Plasma Membrane Is a Molecular Biomarker of Tumor Response to Ionizing Radiation

**DOI:** 10.1371/journal.pone.0012051

**Published:** 2010-08-11

**Authors:** Hailun Wang, Heping Yan, Allie Fu, Miaojun Han, Dennis Hallahan, Zhaozhong Han

**Affiliations:** 1 Department of Radiation Oncology, School of Medicine, Vanderbilt University, Nashville, Tennessee, United States of America; 2 Department of Cancer Biology, School of Medicine, Vanderbilt University, Nashville, Tennessee, United States of America; 3 Department of Radiation Oncology, School of Medicine, Washington University, St. Louis, Missouri, United States of America; 4 Siteman Cancer Center, School of Medicine, Washington University, St. Louis, Missouri, United States of America; 5 Vanderbilt-Ingram Cancer Center, School of Medicine, Vanderbilt University, Nashville, Tennessee, United States of America; Health Canada, Canada

## Abstract

**Background:**

Tumor response to treatment has been generally assessed with anatomic and functional imaging. Recent development of *in vivo* molecular and cellular imaging showed promise in time-efficient assessment of the therapeutic efficacy of a prescribed regimen. Currently, the *in vivo* molecular imaging is limited with shortage of biomarkers and probes with sound biological relevance. We have previously shown in tumor-bearing mice that a hexapeptide (HVGGSSV) demonstrated potentials as a molecular imaging probe to distinguish the tumors responding to ionizing radiation (IR) and/or tyrosine kinase inhibitor treatment from those of non-responding tumors.

**Methodology/Principal Findings:**

In this study we have studied biological basis of the HVGGSSV peptide binding within the irradiated tumors by use of tumor-bearing mice and cultured cancer cells. The results indicated that Tax interacting protein 1 (TIP-1, also known as Tax1BP3) is a molecular target that enables the selective binding of the HVGGSSV peptide within irradiated xenograft tumors. Optical imaging and immunohistochemical staining indicated that a TIP-1 specific antibody demonstrated similar biodistribution as the peptide in tumor-bearing mice. The TIP-1 antibody blocked the peptide from binding within irradiated tumors. Studies on both of human and mouse lung cancer cells showed that the intracellular TIP-1 relocated to the plasma membrane surface within the first few hours after exposure to IR and before the onset of treatment associated apoptosis and cell death. TIP-1 relocation onto the cell surface is associated with the reduced proliferation and the enhanced susceptibility to the subsequent IR treatment.

**Conclusions/Significance:**

This study by use of tumor-bearing mice and cultured cancer cells suggested that imaging of the radiation-inducible TIP-1 translocation onto the cancer cell surface may predict the tumor responsiveness to radiation in a time-efficient manner and thus tailor radiotherapy of cancer.

## Introduction

Radiation therapy, in addition to surgery and chemotherapy, is one of the most commonly prescribed treatments for cancer patients. However, due to the heterogeneity of tumors, not all the tumors respond to a therapy regimen with a similar efficiency. Certain tumors respond to one treatment regimen better than others. The dose and delivery of treatments need to be tailored to each individual patient and progression stage of the tumor [1]. With current assessment approaches that mainly detect the treatment-related anatomic or histological changes within tumors [2], it usually takes weeks to months before the therapeutic response can be detected and the treatment efficacy can be determined. The long clinical delay before a response can be assessed costs patients valuable time on the expensive and potentially ineffective treatments. A time-efficient assessment is especially important in managing the highly malignant lung cancer. For this reason, identification of specific biomarkers for the early assessment of cancer response can help personalized cancer therapy based on the cancer responsiveness to a prescribed regimen.

Imaging the tumor-specific biomarkers has been investigated to monitor the tumor response to treatment [3], [4]. Proteomic and genomic approaches enable new biomarkers discovery by profiling the treatment-associated changes in the abundance of gene transcripts or products [5], [6]. Identification of tumor-specific biomarkers with those approaches relies upon precise sampling and thus requires time- and labor-consuming validations. Compared to the proteomic or genomic techniques, phage display is economic, flexible and easily executed. The i*n vivo* phage display technology [7] takes the advantage of high fidelity without sampling bias and allows identifying the circulation-accessible markers that distinguish tumors from the normal tissues by the spatial location instead of the expression abundance [8]–[10]. Over the past decade, a myriad of phage display-derived peptides have been generated to bind to tumor cells or tumor-associated antigens [10]–[12]. Although phage display-derived peptides show promise in the *in vivo* tumor targeting and molecular imaging of cancer, due to a peptide's small size and relatively low affinity to its molecular target, it is still a great challenge to identify the molecular target that contributes to the peptide binding *in vitro* and *in vivo*. Lack of knowledge about the biological basis of the peptide binding within tumors poses limitations on further development of the tumor-binding peptides for clinical applications.

In a previous study, we have identified a small peptide (HVGGSSV) that specifically binds to the tumors responding positively to ionizing radiation and tyrosine kinase inhibitors by using the *in vivo* phage display [13]. Here, we report that Tax interacting protein 1 (TIP-1, also known as Tax1 binding protein 3, Tax1BP3) is a molecular target of the HVGGSSV peptide, and the radiation-inducible translocation of the predominantly intracellular TIP-1 protein onto the plasma membrane surface serves as a biomarker for the tumor responsiveness to ionizing radiation.

## Materials and Methods

### Cell culture

Lewis Lung Carcinoma (LLC) and H460 lung carcinoma cells were obtained from American Type Culture Collection (ATCC, Rockville, MD, USA) and maintained in DMEM medium with 10% fetal calf serum (FCS) and 1% penicillin/streptomycin (Thermo Scientific Inc., Waltham, MA). Primary human umbilical vein endothelial cell (HUVEC) were obtained from Lonza Biologics (Riverside, CA) and maintained in EGM endothelial cell growth medium. Boyden chambers (Becton Dickinson Labware, Franklin Lakes, NJ) were used to prepare co-culture of HUVEC and cancer cells. Constructs expressing shRNA sequences with green fluorescent protein (GFP) were purchased from Open Biosystems (Thermo Fisher Scientific, Huntsville, AL), the TIP-1 specific shRNA (5′-GGCTAACAGCTGATCCCAA-3′) matches with TIP-1 mRNA transcripts, a non-targeting sequence was used as a control. Transfection of the cells with the recombinant plasmids was conducted with standard protocols [14]. Efficiency of the shRNA on knocking down of TIP-1 expression was detected by western blot analysis of whole cell lysates. Cells were irradiated with 300 kV X-rays using a Pantak Therapax DXT 300 Model X-ray unit (Pantak, East Haven, CT). Antibodies and chemicals were purchased from Sigma (St. Louis, MO) unless otherwise stated.

### Phage Display

Screening [15], [16] of a cDNA library displayed on T7 bacteriophages was employed to identify proteins interacting with the HVGGSSV peptide. In brief, the peptide-immobilized magnetic beads were prepared by incubating 10 µl of 2 mg/ml biotin-GCNHVGGSSV-COOH peptide (Genemed Synthesis Inc., San Antonio, TX) with 100 µl of streptavidin-coated Dynabeads (Invitrogen, Carlsbad, CA) at room temperature for 30 minutes. The same amount of a scramble peptide (Biotin-GCSGVSGHGN-COOH) served as a control in all rounds of the screening. After removal of the free peptides, the beads were resuspended in the phosphate buffered saline (PBS, pH 7.2) containing 0.1% Tween 20 (PBST). The complex was incubated with 10^9^ plaque-forming units (pfu) of a T7Select human lung tumor cDNA library (Novagen, Gibbstown, NJ) in PBS. The mixture was incubated on a shaker for 2 hours at room temperature. The phages bound to the beads were magnetically separated from the unbound phages within solution. After the beads were washed 5 times with PBST, the phages recovered from the HVGGSSV peptide-coated beads were amplified in *E.coli* BLT5615 (Novagen) for the subsequent rounds of screening. In each round of the screening, 10^9^ pfu of the amplified phage were used. The phages recovered from both of the HVGGSSV peptide-coated beads and the control beads were also titrated; selectivity of the phages to the HVGGSSV peptide was estimated with the ratio of the phages recovered from the target beads to those recovered from the control beads. The screening was terminated till the significant (>1000) selectivity of the phages to the target beads was achieved.

The phages recovered from the last round of the screening were cloned and amplified for the enzyme-linked immunosorbent assay (ELISA)-based plate screening. Briefly, 96-well polystyrene plates (Corning Corp., Lowell, MA) were sequentially coated with streptavidin (0.2 µg/well), blocked with 2% BSA in PBS solution, and incubated with the biotinylated HVGGSSV or scramble peptides (50 ng/well). 50 µl of the amplified phages were added to each well for 2 hours incubation at room temperature. After the plates were washed five times with PBST, binding of the phages to the peptides was detected with a rabbit antiserum against the T7 phage (a kind gift from Dr. Toshiyuki Mori at National Cancer Institute, Frederick, MD) and a secondary antibody conjugated with horse radish peroxidase (HRP) (Sigma). Following washing with PBST and incubation with 100 µl of substrate solution containing 2,2′-Azinobis [3-ethylbenzothiazoline-6-sulfonic acid]-diammonium salt (ABTS, from Sigma) within 50 mM sodium citrate buffer, optical density was measured at 405 nm. The insert sequences of the HVGGSSV-specific phage clones were amplified with polymerase chain reactions (PCR) by use of a forward primer (5′- GGAGCTGTCGTATCCAGTC-3′) and a reverse primer (5′-TGGATTGACCGGAAGTAGAC-3′). The PCR products were purified for sequencing reaction by using a sequencing primer (5′-ATGCTCGGGGATCCGAATTC-3′).

### GST-TIP-1 protein production

cDNA encoding TIP-1 was amplified using PCR by use of the selected phage clone as template. A construct encoding TIP-1 mutant with a dysfunctional PDZ domain (H90A) [17] in pcDNA3.1 plasmid was a generous gift from Dr. Paul A. Welling at University of Maryland (Baltimore, MD). The DNA fragments encoding the functional TIP-1 and mutant TIP-1 were respectively subcloned into a pGEX-4T-1 vector (GE Healthcare) between *Bam*H I and *Eco*R I sites to create fusion protein with glutathione S-transferase (GST). The GST-fused proteins were expressed in *E.coli* XL-10 GOLD (Stratagene, Kirkland, WA) and purified to homogeneity by passing through a column packed with GST-binding resin (Thermo Scientific Inc., Waltham, MA)[18]. The purified proteins were dialyzed against PBS and quantified with the Bradford methods [19]. Size and purity of the proteins were examined by SDS-PAGE.

### Peptide binding assay

The ELISA was used to study interaction of the peptide with the recombinant TIP-1 protein. All the biotinylated peptides were synthesized at Genemed Synthesis Inc. (San Antonio, TX), purity and molecular weight were assured with HPLC and mass spectrometry. Corning costar 96-well microtiter plates with high protein-binding capacity were coated with streptavidin (0.2 µg/well in PBS) overnight at 4°C. Plates were blocked with BSA and washed twice with PBST before incubating with the biotinylated peptides (50 ng/well) at room temperature for 1 hour. After washing with PBST, the purified GST-TIP-1 or GST-TIP-1(H90A) proteins were added to each well (100 ng/well), respectively. The recombinant proteins bound to the immobilized peptides were detected by a rabbit anti-GST antibody and a secondary antibody conjugated with HRP (Sigma). After washing with PBST as described above, ABTS solution was added to each well for color development, and optical density at 405 nm was measured for quantification of peptide affinity to the recombinant proteins.

### Preparation of TIP-1 specific antibodies

New Zealand white rabbits (Harlan Laboratories, Prattville, AL) were initially immunized with 100 µg of purified GST-TIP-1 protein premixed in a 1∶1 ratio by weight with Titermax adjuvant (CytRx Corporation, Los Angeles, CA). One month after the initial immunization, the animals were boosted with same amount of antigen twice with 2 weeks interval without the adjuvant. Blood samples were periodically taken for antibody titration and specificity analyses by ELISA or western blot. When the anti-TIP-1 antibody reached the designated high titer, the animals were sacrificed to collect the antiserum. The antiserum was purified by passage through protein A plus protein G columns (sigma) to purify IgGs. TIP-1 specific antibodies were prepared from the purified IgGs by tandem absorption with bacterial proteins and the purified GST protein-conjugated sepharose-4B (Sigma) to remove the IgGs that might bind proteins other than TIP-1. The final antibody was dialyzed against PBS and concentrated *via* Amicon centrifugal filters (Millipore, Billerica, MA). Specificity of the TIP-1 specific antibody was validated with whole cell staining and western blot analyses of whole cell lysates. All animal studies were conducted as approved (protocol ID: M/08/051 and M/08/592) by the Institutional Animal Care and Use Committee (IACUC) at Vanderbilt University.

### Antibody competition assays

*In vitro* antibody competition experiments were performed using the ELISA as described above in the peptide binding assay, except that the purified GST-TIP-1 proteins were pre-mixed with serially diluted antibodies (starting at 10 µg/ml) before incubation with the immobilized peptides. *In vivo* antibody competition assays were conducted as described [13]. In brief, 1×10^6^ LLC cells were implanted subcutaneously in both hind limbs of C57BL/6 Foxn1 null/null nude mice (4∼5 weeks old, Harlan Laboratories). The tumors were allowed to grow to a size of 0.5 cm in diameter before treatment with X-ray radiation (5 Gy) on one tumor while the other tumor in the same mouse was used as an untreated tumor control. All animals were anesthetized and shielded to only allow irradiation of the tumors with 300 kV X-rays using a Pantak Therapax DXT 300 Model X-ray unit (Pantak, East Haven, CT). 200 µg of TIP-1 antibody or control antibody (normal rabbit IgGs) were injected through tail veins 4 hours after the radiation treatment, then followed by injecting 100 µg of Alexa Fluor 750 (Invitrogen)-labeled streptavidin that was pre-complexed with the biotinylated HVGGSSV peptides. Biodistribution of the peptide-strepavidin complex within the tumor-bearing mice was monitored with *In Vivo* Imaging Systems 200 (IVIS 200) (Caliper Life Sciences, Hopkinton, MA) at the Vanderbilt University Institute of Imaging Sciences 24 hours after the peptide injection.

### Animal imaging

Tumor model development, tumor treatment and peptide or antibody labeling with Alexa Fluor 750 (Invitrogen) were conducted as described in previous publication [13]. 20 µg of the fluorochrome-labeled antibody or peptide were intravenously administrated in each animal at 4 hours post irradiation. Optical images were taken at 24 hours post the antibody injection with Xenogen IVIS-200.

### Flow Cytometry

Lung cancer cells LLC and H460, HUVEC, or HUVEC co-cultured with either LLC or H460 (in a Boyden Chamber) were allowed to grow to 80% confluency, and irradiated with 0, 2, 4, 6 or 8 Gy, respectively. At variable time points after the treatment, the cells were detached with accutase (eBioscience, San Diego, CA) and collected by centrifugation at 100× g for 5 minutes. The TIP-1 antibody in PBS containing 2% BSA was added to the suspended cells and incubated on ice for 40 minutes. Free TIP-1 antibody was removed by centrifugation and the cells were resuspended with fresh PBS before Alexa Fluor 488-labeled goat anti-rabbit antibody (Invitrogen) was added and incubated for another 40 minutes on ice. Again the cells were washed and Annexin V-APC (BD Biosciences, Franklin Lakes, NJ) was incubated with the cells for 30 minutes on ice to detect apoptotic cells, Propidium Iodide (PI, BD Bioscience) was added in the last 10 minutes to detect cell plasma membrane integrity. Cells were scanned with a BD LSRII flow cytometer (BD bioscience).

TIP-1-positive and -negative H460 cells were sorted from the PI-negative cell populations with the FACSAria (BD bioscience) for proliferation and radiosensitivity assays. The cell proliferation was surveyed with Ki-67 staining. Briefly, the sorted TIP-1 -positive or -negative cells were cultured on glass coverslips at 37°C for 24 hours. The cells were then fixed in 70% ethanol, permeated with 0.5% Triton X-100, and stained with Ki-67 antibody (Vector laboratories, Burlingame, CA). Cells were counterstained with DAPI (4′,6-diamidino-2-phenylindole) before examination under fluorescence microscope. The susceptibility of the TIP-1-positive and -negative H460 cells to ionizing radiation was evaluated with a clonogenic assay in which the sorted cells were seeded and cultured with DMEM medium supplemented with 10% FCS and antibiotics in petri dishes for 4 hours before the cells were irradiated with X-ray at variable doses. The colonies were allowed to form in 12 days. The colonies were stained with methylene blue solution and the colony numbers were counted for data analyses.

### Statistical analyses

All data were analyzed with the Student's t-test to determine significance (with >95% confidence) of the differences. Results are presented as mean ± S.D.

## Results

### TIP-1 binds to the HVGGSSV peptide

We have previously shown that one phage display-derived peptide HVGGSSV with the selective binding within a broad spectrum of tumors including lung cancers that had been treated with X-ray radiation and multiple tyrosine kinase inhibitors, the peptide binding within the treated tumors correlated to the overall efficacy of the treatment on the tumor growth control [13]. To further develop this peptide for molecular imaging and tumor-targeted drug delivery, we intended to identify the molecular target that enables the selective binding of the HVGGSSV peptide within irradiated tumors. Screening a T7 phage-displayed human lung tumor cDNA library against the HVGGSSV peptide revealed TIP-1 as one protein that enables the selective binding pf the HVGGSSV peptide within irradiated tumors.

**Figure 1:**
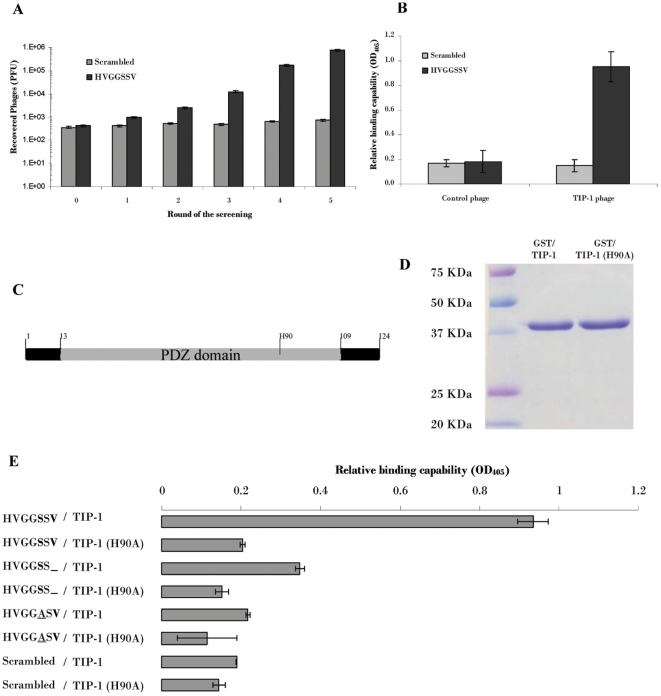
HVGGSSV peptide binds to PDZ domain of the TIP-1 protein. **A**: Enrichment of phages with selective binding to the HVGGSSV peptide. Phages recovered from the five rounds of screening and those from the original phage library were subjected to selectivity analysis on beads coated with the HVGGSSV peptide or a scrambled peptide control, respectively. Shown are number (PFU) of phages recovered from the beads after the free phages were washed off. 10^9^ PFU of phages were used in this assay. **B**: Specificity of the TIP-1-expressing phage to the HVGGSSV peptide. **C**: Diagram of TIP-1 protein shows location of PDZ domain and the critical amino acid (H90) for PDZ ligand binding; **D**: SDS-PAGE image shows purity of the recombinant TIP-1 proteins and a dysfunctional mutant TIP-1 (H90A). The fusion proteins (∼37 kD) were analyzed along with molecular weight markers. **E**: Relative association of the purified recombinant proteins to the synthetic peptides was evaluated with ELISA. In these assays, 100 ng of the purified GST/TIP-1 or GST/TIP-1(H90A) proteins were used per wells, all the synthetic peptides were used as 50 ng per well. The mutations in the PDZ binding motif were underlined. Shown are representative data from triplicate experiments. * *p*<0.05, *n* = 3, the Student's *t*-test.

To identify the molecular target of the HVGGSSV peptide within irradiated tumors, a T7 phage-displayed human lung tumor cDNA library composed of 10^7^ independent phage clones was screened against the HVGGSSV peptide that was immobilized onto magnetic beads. The beads with a scramble peptide were used as a control to deplete the non-specific phage clones. The phages recovered from the HVGGSSV peptide-coated beads, as well those recovered from the control beads, were titrated by following procedures as described [15], [16]. After 5 rounds of such screening, it was found that total phages showed more than 1000-fold selectivity to the HVGGSSV peptide-coated beads over the control beads (Fig. 1A), indicating the screening successfully enriched some phage clones that selectively bind to the HVGGSSV peptide. Single clones from the fifth round of the biopanning were isolated for further ELISA-based plate screening. Roughly 46.8% (22/47) of the analyzed phage clones exhibited specific binding to the HVGGSSV peptide but no detectable binding was observed to the control peptide which has same amino acid composition but with a scrambled amino acid sequence (data not shown). 17 out of the 20 (85%) sequenced HVGGSSV-binding phage clones encoded amino acid sequence of the TIP-1 [20]. Specificity of the TIP-1 expressing phages to HVGGSSV peptide was shown (Fig. 1B) in the ELISA-based assays.

### TIP-1 binds to the HVGGSSV peptide via PDZ domain

To further study interaction of the HVGGSSV peptide and TIP-1 protein, gene fragments encoding TIP-1 protein were inserted into an expression plasmid to produce recombinant GST-TIP-1 proteins in *E. coli*. The GST tag was utilized to purify the recombinant proteins to homogeneity (Fig. 1D). Affinity of the HVGGSSV peptide with TIP-1 protein was studied with the ELISA by use of the purified recombinant proteins and synthetic peptides (Fig. 1E). TIP-1 protein is a small protein of 124 amino acids, a PDZ-domain is the only functioning structure identified so far (Fig. 1C). The HVGGSSV peptide contains a canonical PDZ binding motif (-X-S/T-X-V/L/I, whereas X represents any hydrophobic amino acids) [21], the peptide probably binds to TIP-1 through the PDZ domain. Therefore a TIP-1 mutant protein (H90A) with abolished PDZ ligand binding capability [17] was included in the binding assay. Several peptides with mutation within the PDZ binding motif were also included in the ELISA-based affinity study. The data showed that the HVGGSSV peptide binds to the TIP-1 protein through the PDZ domain, it can not bind to the H90A mutant which contains a dysfunctional PDZ domain. The scramble peptide, as well as other peptides with single mutation within the PDZ binding motif, did not show significant binding to the TIP-1 protein (Fig. 1E).

### TIP-1 specific antibody competes with HVGGSSV peptide for TIP-1 binding

**Figure 2:**
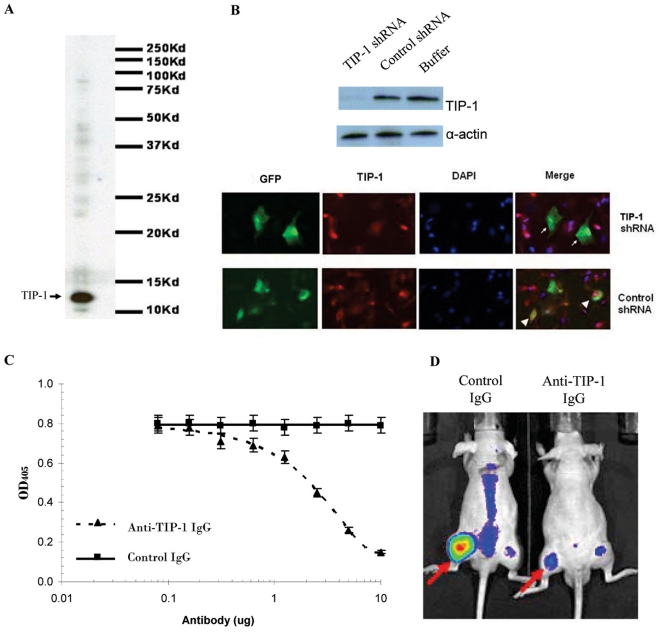
TIP-1 specific antibody blocked the HVGGSSV peptide binding within irradiated tumor. **A**: Specificity of the TIP-1 antibody as revealed with western blot analysis of whole LLC cell lysate. The endogenous TIP-1 protein (∼14 kD) recognized by the antibody was pointed with arrow. **B**: Specificity of the TIP-1 antibody as demonstrated in immunofluorescent staining of LLC cells that were transfected with shRNA plasmids. Effect of the TIP-1 targeting shRNA on TIP-1 expression was determined with western blot analysis (upper panel). In the cell staining, the transfected cells were tracked with GFP protein expression from the shRNA plasmids. TIP-1 was stained as red with the TIP-1 antibody. Cell nuclei were stained with DAPI. The LLC cells transfected with the control shRNA that did not affect TIP-1 expression were pointed with arrow head, while the cells transfected with TIP-1 targeting shRNA that abolished TIP-1 expression were pointed with arrows (lower panel). **C**: ELISA-based *in vitro* competition assay. Serially diluted antibodies were respectively pre-incubated with the purified GST/TIP-1 proteins (100 ng/well) before the complex was added to the plates coated with the HVGGSSV peptide (50 ng/well). The GST/TIP-1 protein associated to the immobilized HVGGSSV peptide was detected with GST-specific antibody. **D**: Optical images of LLC tumor-bearing mice that were co-administrated with the Alexa Fluor 750-labeled HVGGSSV peptide and antibodies. LLC tumors in the left hind limbs were irradiated at 5 Gy (pointed with arrows). 200 µg of the TIP-1 antibody, or the control antibody, was injected at 2 hours post the IR treatment, followed by injection of Alexa Fluor 750-labeled HVGGSSV peptide at 4 hours post the IR treatment. Optical images were acquired 24 hours after the peptide injection. The presented data represent three independent experiments.

TIP-1 antibody was developed by immunizing rabbits with the purified GST-TIP-1 proteins. Specificity and reactivity of the TIP-1 antibody were determined by western blot analysis of whole LLC cell lysate and immunofluorescent staining of LLC cells in which TIP-1 expression had been depleted with specific shRNA. The TIP-1 specific antibody only recognized a single band corresponding to the endogenous TIP-1 protein (∼14 kD) in a western blot analysis of the LLC whole cell lysates, with minor or undetectable binding to other unrelated proteins (Fig. 2A). Cell staining further demonstrated specificity of the TIP-1 antibody. We identified one out of a panel of shRNA constructs that efficiently down-regulated TIP-1 expression within LLC cells, as shown by western blot analysis of whole cell lysate (upper panel of Fig. 2-B). This TIP-1 targeting shRNA was selected for transfection of LLC cells and the transfection was tracked with GFP expression from the shRNA plasmid. Overlapping of the TIP-1 antibody (red) and GFP (green) was observed within the LLC cells that were transfected with a construct with a control shRNA (pointed with arrow heads). The TIP-1 specific antibody did not stain the LLC cells that were transfected with a construct with the TIP-1-targeting shRNA (pointed with arrows), because the target of the antibody had been depleted with the TIP-1 targeting shRNA (lower panel of Fig. 2B). These data demonstrated that the TIP-1 antibody did not bind to any protein other than TIP-1 within the cancer cells. These results confirm that this is a TIP-1 specific antibody.

The TIP-1 specific antibody inhibits the HVGGSSV peptide binding to the recombinant TIP-1 protein in a dose-dependent manner as measured by ELISA (Fig. 2C), whereas the control antibody did not interfere with the binding. The results of these experiments demonstrate that the TIP-1 specific antibody share or overlap with the HVGGSSV peptide for a common interacting areas within the TIP-1 protein. Therefore, this TIP-1 antibody was used to study the contribution of TIP-1 to the selective binding of the HVGGSSV peptide within irradiated tumors.

### TIP-1 mediates binding of the HVGGSSV peptide within irradiated tumors

To determine if TIP-1 binds to the HVGGSSV peptide within irradiated tumors, antibodies were intravenously administrated in LLC tumor-bearing mice prior to the injection of the fluorophore-labeled HVGGSSV peptide. Optical imaging data showed that binding of the HVGGSSV peptide within the irradiated tumors was not affected by pre-injection of the control IgGs. However, pre-injection of the TIP-1 specific IgGs dramatically attenuated the accumulation of the fluorophore-labeled peptide within the irradiated tumors (Fig. 2D). These data clearly demonstrated that, at least in part, TIP-1 mediates the selective binding of the HVGGSSV peptide within the irradiated tumors.

### TIP-1 specific antibody exhibited similar binding patterns as the HVGGSSV peptide within irradiated tumors

**Figure 3:**
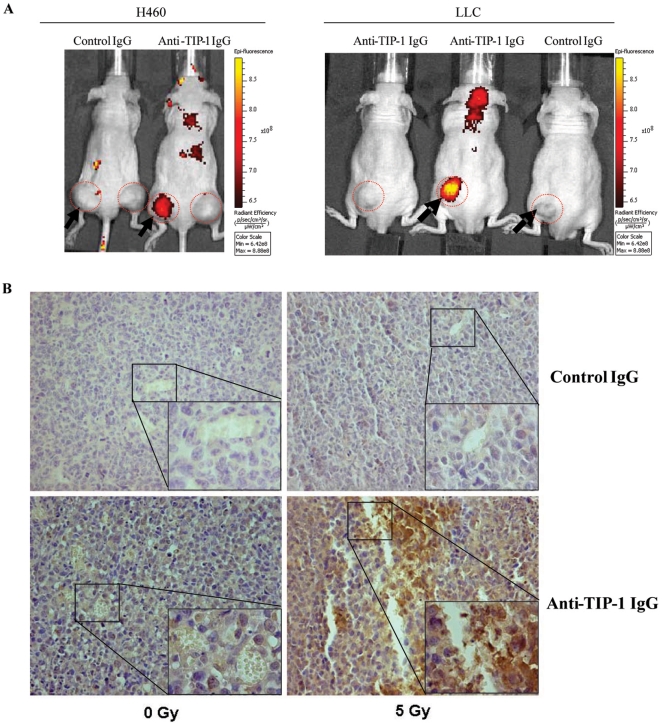
TIP-1 specific antibody was selectively homed to the irradiated tumors. **A**: NIR images of the TIP-1 antibody distribution within tumor-bearing mice. H460 and LLC tumors were developed in hind limbs of nude mice, respectively. Tumors were indicated within the dashed circles. The irradiated tumors (at 5 Gy) were pointed with arrows. Alexa Fluor 750-labeled control or TIP-1 specific antibodies were injected 4 hours after the irradiation. The showed representative optical images were acquired 24 hours after the antibody injection. **B**: Immunohistochemical staining of the intravenously administrated antibodies within the irradiated tumors. The imaged tumors were retrieved for detection of the antibodies within the tumors. The antibodies (rabbit IgGs) were detected with HRP-conjugates and visualized with DAB (shown as brown). Hematoxylin (blue) was used for counterstaining. Representative images from H460 tumor sections were presented, high magnification (x400) images were shown as inserts.

We have previously shown that HVGGSSV peptide specifically binds to the tumors responding positively to radiation and/or tyrosine kinase inhibitors [13]. If TIP-1 contributes to the peptide accumulation within irradiated tumors, one logic prediction is that the TIP-1 antibody can recapitulate the biodistribution pattern of the HVGGSSV peptide in tumor-bearing mice. To test this hypothesis, nude mice bearing H460 or LLC xenografts were irradiated, Alexa Fluor-750 labeled TIP-1 antibody was injected *via* tail veins 4 hours after the radiation treatment by following the same protocol that was used to study the biodistribution of the HVGGSSV peptide within tumor-bearing mice [13]. Optical images acquired 24 hours after the antibody injection indicated that the TIP-1 antibody had high selectivity to the irradiated tumors, but not the untreated tumors or normal tissues in both of the LLC and H460 tumor models (Fig. 3A). In this regard, accumulation of the TIP-1 specific antibody within the irradiated tumors was confirmed with immunohistochemical staining (Fig. 3B) of the retrieved tumor tissues after animal imaging.

### Radiation induces TIP-1 translocation onto the plasma membrane surface

**Figure 4:**
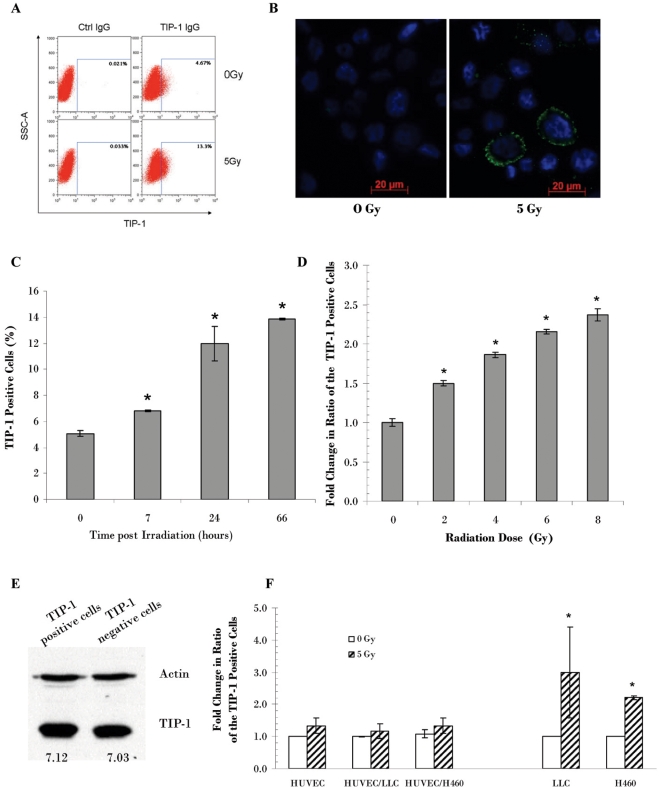
Radiation induced TIP-1 translocation onto the plasma membrane of the cancer cells. **A**: Flow cytometric profile of TIP-1 expression on the H460 cell surface. TIP-1 on the cell surface was detected with the TIP-1 antibody 24 hours after radiation treatment, a control IgG was included to demonstrate the antibody specificity. **B**: Fluorescent staining of the TIP-1 expression (green) on the cell surface of the irradiated H460 cells. DAPI was used for counterstaining. **C**: Time course study. The H460 cells were irradiated at 5 Gy and then fixed at variable time points after irradiation for flow cytometric analysis of the TIP-1 expression on the cell surface. Percentage of the TIP-1 positive cells was presented. **D**: The dose-dependence study. The H460 cells were irradiated with variable dose of X-ray, the cells were fixed 24 hours post the irradiation for profiling the TIP-1 expression on the cell surface with flow cytometry. Fold change of the TIP-1 positive cells was calculated by comparison to the untreated cells (counted as 1). **E**: Western blot analysis of TIP-1 expression within the TIP-1 positive or negative cells that were sorted from the irradiated (5 Gy) H460 cells 24 hours after the irradiation. Relative TIP-1 protein level was normalized to that of the actin control (counted as 1) and shown under the image. **F**: Flow cytometric profile of TIP-1 expression on the cell surface of LLC, H460 or HUVEC cells. The cells were treated with 5 Gy of X-ray, TIP-1 on the cell surface was profiled 24 hours post the radiation treatment. Fold change of the TIP-1 positive cells was calculated by comparison to the untreated cells (counted as 1). * *p*<0.01, *n* = 3, the Student's *t*-test, each was compared to the untreated control, respectively.

TIP-1 is a basically intracellular protein that is ubiquitously expressed within multiple organs [22]. The tumor-specific and radiation-inducible binding the HVGGSSV peptide and the TIP-1 antibody within tumor-bearing mice suggested that TIP-1 accessibility to the circulating peptide or antibody is inducible upon radiation treatment and limited within tumor cells. We used cultured LLC, H460 lung cancer cells and HUVEC cells to study the IR-induced TIP-1 translocation onto the cell surface. Western blot analysis of the whole cell lysates did not show dramatic changes in the TIP-1 protein level after IR in all the three tested cells (data not showed). However, flow cytometric analysis showed that percentage of the H460 cells with TIP-1 expression on the cell surface was elevated from basal level (untreated cells) of 4.67% to 13.3% in 24 hours after IR treatment (Fig. 4A). This observation was supported by immunofluorescent staining of the surface-located TIP-1 on the irradiated H460 cells (Fig. 4B). It was found that the translocation of the TIP-1 protein onto the H460 cell plasma membrane surface sustained for a prolonged period of time after the irradiation (Fig. 4C). The effect of IR on TIP-1 translocation is dose-dependent (Fig. 4D), higher dose of IR was more effectively inducing the TIP-1 translocation onto the cell surface. A similar effect of radiation on the TIP-1 expression on the cell surface were also observed on the LLC cells, but not the HUVEC cells even the endothelial cells had been co-cultured with LLC or H460 cancer cells (Fig. 4F). These data suggested that radiation induced translocation of the intracellular TIP-1 onto the plasma membrane might be limited to cancer cells.

The TIP-1 positive and negative H460 cells were sorted for western blot analysis to determine whether they represent two independent subgroups of cells with different TIP-1 protein level. The result showed that the total TIP-1 levels were similar in both of the TIP-1 positive and negative cells (Fig. 4E). Combining with the observations that IR did not significantly change overall TIP-1 protein levels in the tested cells, we concluded that radiation does not alter total protein levels of TIP-1 but rather appears to promote the TIP-1 translocation onto the plasma membrane surface.

### The radiation-induced TIP-1 translocation relates to the reduced colony formation and proliferation potentials and the increased susceptibility to subsequent radiation treatment

**Figure 5:**
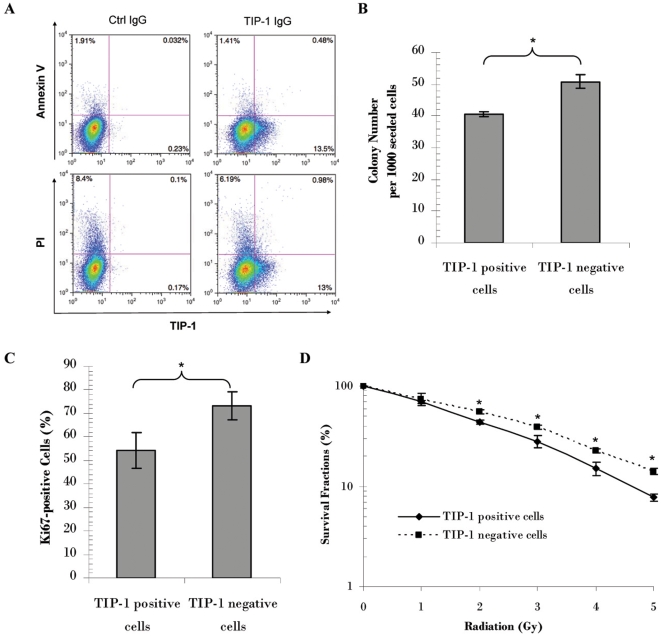
The TIP-1 translocation onto the cell surface is one biomarker other than those for the cell death and apoptosis. **A:** Flow cytometric profile of TIP-1 expression on the cell surface, apoptosis (Annexin V) and cell death or membrane integrity (PI) at 24 hours after the radiation (5 Gy) treatment of the H460 cells. Percentage of the cells in each section was shown in the representative profile images from three independent experiments. **B**: Colony formation on petri dishes of the TIP-1 positive and negative cells sorted from the irradiated H460 cells. The H460 cells were irradiated at 5 Gy. The TIP-1 positive and negative cells were sorted 24 hours post the radiation treatment. Colony formation capability of the two subgroups of cells was shown as colony number per 1000 seeded cells. **C**: Proliferation capability of the sorted cells as determined with Ki-67 staining. Percentage of the proliferative cells among the counted cells was shown. **D**: Susceptibility of the sorted H460 cells to subsequent irradiation. Shown are data from clonogenic assays. Survival fractions were presented to show difference of the radiation susceptibility between the TIP-1 positive and negative cells. * *P*<0.05, *n* = 3, the Student's *t*-test.

Annexin V profiling (apoptosis) and PI staining (membrane integrity and cell death) are commonly used to detect cellular response to cytotoxic treatment. H460 cells were irradiated (5 Gy) and dissociated 24 hours after the irradiation for flow cytometric analysis. Triple color flow cytometric profiling showed that majority of cells with TIP-1 expressed on the cell surface (TIP-1 positive cells) are negative for both of the dead cell marker (PI) and the apoptotic cell marker (Annexin V) (Fig. 5A). Among the total apoptotic cells (0.48%+1.41%  = 1.89%), only one third (0.48% out of 1.89%) of them were stained as TIP-1 positive. Among the TIP-1 positive cells (13.5%+0.48% = 13.98%), majority (13.5% out of 13.98%) were Annexin V negative. Among all the dead cells (0.98%+6.19% = 7.17%), only small portion (0.98% out of 7.17%) of the dead cells were stained as TIP-1 positive. Among all the TIP-1 positive cells (13%+0.98% = 13.98%), majority (13% out of 13.98%) were PI-negative. These data suggested that radiation-inducible TIP-1 translocation onto the cell surface does not overlap with the treatment associated apoptosis or cell death.

The TIP-1 positive and negative H460 cells were sorted from the irradiated H460 cells for *in vitro* colony formation, proliferation and radiation susceptibility studies. Although both of irradiated TIP-1 positive and negative cells showed very low capability to form visible colonies on petri dishes, statistical significance was observed between the two subgroups of cells in respect of the capability of colony formation (Fig. 5B). Ki-67 staining (Fig. 5C) further showed that fewer TIP-1-positive cells were undergoing proliferation than the TIP-1-negative cells. Clonogenic assays (Fig. 5D) indicated that fewer TIP-1 -positive cells survived after subsequent radiation treatment than the TIP-1 -negative cells. All these data suggested that the radiation-inducible TIP-1 translocation onto the cancer cell surface serve as a biomarker for identifying radiation-responding cancers before the onset of apoptosis and cell death.

## Discussion

Not all tumors, not even within the same classifications, respond to a treatment in a same way. Personalized or tailored treatment of tumor calls for efficient and reliable assessment of the tumor responsiveness. Even though anatomic and functional imaging have been extensively investigated and applied to assess tumor response to treatment, new biomarkers with sound biological relevance are still needed to assess tumor response to treatment in a time-efficient manner.

In an effort to identify such biomarkers, we previously identify a short peptide (HVGGSSV) with *in vivo* phage display technology. The peptide demonstrated potentials in assessing the tumor responsiveness to radiation and tyrosine kinase inhibitors at the early stage of treatment courses [13]. As demonstrated within multiple heterotopic and orthotopic tumor models, the peptide selectively binds to the responding tumors, the peptide accumulation within the treated tumors correlates to the overall biological effects of the treatment on the tumor growth control. In this study, TIP-1 was identified as one molecular target of the HVGGSSV peptide. TIP-1 specific antibody competed with the HVGGSSV peptide for binding within irradiated tumors, and exhibited similar binding patterns as the peptide in tumor-bearing mice. It was further identified that radiation induced translocation of the basically intracellular TIP-1 protein onto the cell surface in a dose-dependent manner. The treatment-induced TIP-1 expression on the cell surface is detectable in the first few hours after the treatment and before the onset of treatment associated apoptosis or cell death. In fact, majority of the cells expressing TIP-1 on the cell surface are the live but still responding cancer cells, albeit such cells are less potent in proliferation and more susceptible to subsequent radiation treatment. Although it still under investigation to understand the mechanism and biological consequence of the radiation-induced TIP-1 translocation, these data support one conclusion that the radiation-inducible translocation of TIP-1 onto the cell surface holds promise as one surrogate biomarker in assessing the tumor responsiveness to ionizing radiation.

Discovery of the TIP-1 translocation onto the cell surface as one biomarker of tumor response to radiation took advantages of phage display technologies. Firstly, a peptide HVGGSSV was identified with selective binding to the tumors responding to IR and tyrosine kinase inhibitors with *in vivo* phage display [13]. Cancer cell distinguishes itself from the normal cell by expressing proteins or receptor on the cell surface, imaging of such surface proteins such as EGFR has been studied to track the tumor progression or even monitor tumor response to treatment [4]. We envision that the treatment-inducible protein expression on the cancer cell surface holds promise as surrogate biomarkers for imaging-based assessment of the tumor responsiveness to treatment. Compared to the genomic and proteomic profiling [23] that focus on the gene structure and overall expression abundance, *in vivo* phage display prefers the molecule that are localized on the cell surface and circulation accessible. Moreover, unlike the subcellular proteomic profiling [24] that has been explored in biomarker discovery *in vivo*, the *in vivo* phage display takes advantages of the minimal sample bias and the real time *in vivo* binding within the sophisticated tissues and cell structures. Secondly, TIP-1 was identified as the molecular target of the HVGGSSV peptide through biopanning a phage-displayed cDNA library. In the effort to identify the molecular target(s) of the HVGGSSV peptide, no meaningful data was generated through BLAST search for homologous sequences [11], affinity purification accompanied with mass spectrometric identification [9], or yeast-two hybrid screening of cDNA libraries [8]. Low affinity or low abundance of the corresponding molecular target(s) might contribute to the difficulty in identification of the molecular target(s) of the short peptide. A phage-displayed cDNA library was screened against the HVGGSSSV peptide, rounds of biological amplification and affinity selection significantly enriched the peptide-binding clones that lead to the TIP-1 identification. This study further demonstrated the potentials of screening phage-displayed cDNA library in discovery of molecular targets of the peptides with simple structure and low affinity.

TIP-1 is ubiquitously expressed within multiple organs [25]. It is predominantly localized in the cytoplasma [25], [26], with rare or undetectable expression in the nucleus or on the cell plasma membrane under normal culturing condition. It has been studied as a PDZ antagonist in modulating cell proliferation, polarity, migration and stress response [17], [ 22], [ 26], [ 27]. However, its biological functions in cancer biology and cell stress response are still under investigation. Our flow cytometry and cell imaging data showed that the TIP-1 translocation onto the cell surface after X-ray irradiation was dominantly observed in cancer cells, but did not extend to endothelial cells as tested in this study by the use of HUVEC (Fig. 4F). This difference is not related to the abundance of the TIP-1 protein within the cells. Western blot analysis of TIP-1 expression within the whole cell lysates indicated that TIP-1 was expressed in all the cell lines including the HUVEC with comparable protein level (data not shown). Tissue staining also showed that the intravenously administrated TIP-1 antibody was dominantly associated to the tumors cells (Fig. 3B). These data suggested that the radiation-induced TIP-1 translocation onto the cell surface might be limited to the tumor cells. This conclusion is also supported by our previous observations that the HVGGSSV peptide did not bind to normal tissues that had been irradiated or inflamed with LPS and TNF-α [13]. Contradictorily, radiation-induced translocation of other intracellular proteins such as P-Selectin [28] and intercellular adhesion molecule-1 (ICAM-1) [29] are reportedly associated with inflammatory response to ionizing radiation and thus not specific to tumor response to the radiation treatment. In this regard, the radiation-induced TIP-1 translocation onto the cancer cell surface is one unique biomarker of tumor response to radiation.

Although further investigation is needed to elucidate the mechanism(s) by which X-ray irradiation induces the TIP-1 translocation onto the cell surface and the biological relevance of the TIP-1 translocation in the tumor response to radiation, we revealed that the cells responding to radiation by relocating TIP-1 onto the cell surface are live but have reduced capability to proliferate and form colonies. The cells with TIP-1 expression on the cell surface are more susceptible to subsequent radiation treatment, compared to the counterparts of the cells without TIP-1 expression on the cell surface. These data partially explains why TIP-1 imaging with the HVGGSSV peptide is predictive in assessing the tumor responsiveness to radiation [13]. The TIP-1 translocation was detectable in the first few hours after radiation treatment and before the onset of the treatment associated apoptosis and cell death, suggesting a potential mechanism to assess tumor response to IR at an early time point of a treatment course.
